# Editorial: The Potential of Machine Learning in Pharmacogenetics, Pharmacogenomics and Pharmacoepidemiology

**DOI:** 10.3389/fphar.2022.928527

**Published:** 2022-05-20

**Authors:** Augusto Garcia-Agundez, Elena García-Martín, Carsten Eickhoff

**Affiliations:** ^1^ Brown University, Providence, RI, United States; ^2^ Universidad de Extremadura, University Institute of Molecular Pathology Biomarkers, ARADyAL Instituto de Salud Carlos III, Caceres, Spain

**Keywords:** machine learning, artificial intellegence, pharmacogenetics, pharmacoepidemiology, pharmacogenomics

Recent advances in deep learning, natural language processing, and information retrieval show great potential for enhancing the knowledge and processes in the fields of pharmacogenetics, pharmacogenomics, and pharmacoepidemiology. These techniques allow for the unprecedented analysis of large unstructured datasets that would otherwise be intractable, such as finding relevant patterns and clusters in the data being analyzed (e.g. free-text notes in Electronic Health Records, or Pubmed articles or abstracts). From predicting patient outcomes and guiding drug choices that prevent adverse reactions to drug discovery and repurposing, artificial intelligence holds great promise when it comes to the development of support systems to aid clinical decision making in the coming years.

In this Frontiers Research Topic, we invited potential contributors to submit their work and latest advances in deep and machine learning applied to pharmacogenetics, pharmacogenomics, and pharmacoepidemiology.

One of the potential applications of ML in pharmacogenomics is to improve dose prediction accuracy, improving outcomes and reducing adverse drug events (ADEs). In this context, Steiner et al. employ regression to predict stable warfarin dosages in a diverse cohort that includes US Latinos and Latin Americans, concluding that the inclusion of ethnicity and warfarin indication, in addition to the International Warfarin Pharmacogenetics Consortium’s recommended set of variables can result in a small but significant improvement in correct dose prediction.

Also in the field of ADE avoidance, Kang et al. aim to predict the risk of fetal teratogenicity caused by prescribed antiseizure medications (ASMs), which is especially relevant in novel ASMs where prior evidence is limited. To this end, they employ a Support Vector Machine on a series of curated FDA-approved ASMs with risk for teratogenicity, using data from the FDA Spontaneous Adverse Events Reporting System (FAERS) as ground truth and outperforming state-of-the-art approaches.

Pandi et al. propose text-mining to automatically compute potential pharmacogenomic associations. To achieve this, PubMed abstracts and articles are selected, PubTator Central annotations are used for entity recognition. Extracted sentences are then used to extract genome-chemical pairs using different classifiers.

Finally, Fusaroli et al. propose Adversome, a new approach to detect ADEs and other disease- and comorbidity-related syndromes in rapidly changing and low-resource environments such as the COVID epidemic. This network also uses FAERS as their data source to better pinpoint ADEs caused by drugs repurposed for COVID, better adapted than conventional approaches in the aforementioned context.

We are happy to announce that this Research Topic has been re-launched and we look forward to new contributions in the coming months.

